# Improving multi-population genomic prediction accuracy using multi-trait GBLUP models which incorporate global or local genetic correlation information

**DOI:** 10.1093/bib/bbae276

**Published:** 2024-06-10

**Authors:** Jun Teng, Tingting Zhai, Xinyi Zhang, Changheng Zhao, Wenwen Wang, Hui Tang, Dan Wang, Yingli Shang, Chao Ning, Qin Zhang

**Affiliations:** Shandong Provincial Key Laboratory of Animal Biotechnology and Disease Control and Prevention, College of Animal Science and Technology, Shandong Agricultural University, Tai’an 271018, Shandong, China; Shandong Futeng Food Co. Ltd., Zaozhuang 277500, Shandong, China; National Key Laboratory of Wheat Improvement, College of Life Science, Shandong Agricultural University, Tai’an 271018, Shandong, China; Shandong Provincial Key Laboratory of Animal Biotechnology and Disease Control and Prevention, College of Animal Science and Technology, Shandong Agricultural University, Tai’an 271018, Shandong, China; Shandong Provincial Key Laboratory of Animal Biotechnology and Disease Control and Prevention, College of Animal Science and Technology, Shandong Agricultural University, Tai’an 271018, Shandong, China; Shandong Provincial Key Laboratory of Animal Biotechnology and Disease Control and Prevention, College of Animal Science and Technology, Shandong Agricultural University, Tai’an 271018, Shandong, China; Shandong Provincial Key Laboratory of Animal Biotechnology and Disease Control and Prevention, College of Animal Science and Technology, Shandong Agricultural University, Tai’an 271018, Shandong, China; Shandong Provincial Key Laboratory of Animal Biotechnology and Disease Control and Prevention, College of Animal Science and Technology, Shandong Agricultural University, Tai’an 271018, Shandong, China; College of Veterinary Medicine, Shandong Agricultural University, Tai’an 271018, Shandong, China; Shandong Provincial Key Laboratory of Animal Biotechnology and Disease Control and Prevention, College of Animal Science and Technology, Shandong Agricultural University, Tai’an 271018, Shandong, China; Shandong Provincial Key Laboratory of Animal Biotechnology and Disease Control and Prevention, College of Animal Science and Technology, Shandong Agricultural University, Tai’an 271018, Shandong, China

**Keywords:** multi-population, genomic prediction, MTGBLUP, global genetic correlation, local genetic correlation

## Abstract

In the application of genomic prediction, a situation often faced is that there are multiple populations in which genomic prediction (GP) need to be conducted. A common way to handle the multi-population GP is simply to combine the multiple populations into a single population. However, since these populations may be subject to different environments, there may exist genotype-environment interactions which may affect the accuracy of genomic prediction. In this study, we demonstrated that multi-trait genomic best linear unbiased prediction (MTGBLUP) can be used for multi-population genomic prediction, whereby the performances of a trait in different populations are regarded as different traits, and thus multi-population prediction is regarded as multi-trait prediction by employing the between-population genetic correlation. Using real datasets, we proved that MTGBLUP outperformed the conventional multi-population model that simply combines different populations together. We further proposed that MTGBLUP can be improved by partitioning the global between-population genetic correlation into local genetic correlations (LGC). We suggested two LGC models, LGC-model-1 and LGC-model-2, which partition the genome into regions with and without significant LGC (LGC-model-1) or regions with and without strong LGC (LGC-model-2). In analysis of real datasets, we demonstrated that the LGC models could increase universally the prediction accuracy and the relative improvement over MTGBLUP reached up to 163.86% (25.64% on average).

## Introduction

Genomic prediction (GP), also known as genomic selection (GS), has been widely applied in animal [1, 2] and plant breeding [3, 4] and has tremendously accelerated the genetic gain of almost all major breeding goal traits because of its higher prediction accuracy.

In the application of GP, a situation often faced is that there are multi-populations in which GP need to be conducted. Although GP can be conducted within each population, it is often difficult to form a larger reference population for some populations due to the limited size of active breeding population. Furthermore, a cross-population GP, i.e. using a reference population from one population, which is large, for GP in another population, is not feasible if they may have different genetic backgrounds [5–7]. A commonly used approach for multi-population GP is to set up a joint reference population by combining individuals from multi-populations and then use this reference population for GP in these populations. This approach has been proved to be effective and could potentially increase prediction accuracy in comparison to within population GP [8–10]. However, there are two problems related to this approach: first, in some cases, the trait of interest is not defined or measured in the same way in different populations, so it is not reasonable to combine these population together; second, these populations are subject to different environments and there may exist genotype by environment (G × E) interaction, which may reduce the prediction accuracy [11, 12]. Following the idea of Falconer [13] that the performances of a trait in different environments can be regarded as different traits, the G × E interaction can be addressed by using genetic correlations. Based on this idea, multi-population prediction can be regarded as multi-trait prediction using a multi-trait model, like multi-trait genomic best linear unbiased prediction (MTGBLUP). In comparison to simply combining multiple populations, the multi-trait model does not suffer from the two problems mentioned above and thus is flexible for multi-population prediction. Several studies have demonstrated the advantages of MTGBLUP in multi-population GP [14–16].

In a conventional multi-trait model, the genetic correlation used is actually global genetic correlation. Genetic correlations between traits are primarily induced by pleiotropic effects of genes. Since complex traits are usually influenced by a large number of genes, genetic correlations are attributed by many genes distributed in different regions of the genome. Thus, genetic correlations can be regarded as the integration of many regional or LGC, i.e. correlation caused by genes in certain regions. With the rapid increased availability of large-scale sequencing data, it is now feasible to estimate LGCs using regional genetic variants. LGCs have been extensively studied in human genetic analysis [17–19]. These studies proved that LGC could be used to quantify the genetic similarity of complex traits in specific genomic regions and thus to reveal the shared biological mechanisms across traits. Recently, Miao et al. [20] proposed a statistical framework called X-Wing that incorporates the between-population LGCs to identify the shared genetic basis of phenotypes between populations, leading to improved accuracy in polygenic risk score prediction for 31 traits in East Asians. Therefore, it is expected the conventional multi-trait GP model can be improved by replacing global genetic correlation by LGCs and implemented for multi-population GP.

In this study, we present two novel methods for integrating between-population LGC information into multi-population GP. Our methods are based on the MTGBLUP framework and involve grouping single nucleotide polymorphisms (SNPs) according to the significance or degree of LGCs of the regions in which they are located. To assess the effectiveness of our methods, we conducted extensive benchmarking using real datasets from mice and cow populations. Through comprehensive analyses, we demonstrate the superior performance of the proposed methods compared to conventional multi-population prediction method (MTGBLUP).

## Methods

### Heritability and global genetic correlation estimation

For the trait of interest, which is phenotyped in two populations, a bivariate linear mixed model was used to estimate its heritabilities within the two populations and the global genetic correlation between the two populations. The model is as follows,


(1) \begin{equation*} \left[\begin{array}{@{}c@{}}{\mathrm{y}}_1\\{}{\mathrm{y}}_2\end{array}\right]=\left[\begin{array}{@{}cc@{}}{\mathrm{X}}_1& 0\\{}0& {\mathrm{X}}_2\end{array}\right]\left[\begin{array}{@{}c@{}}{\mathrm{b}}_1\\{}{\mathrm{b}}_2\end{array}\right]+\left[\begin{array}{@{}cc@{}}{\mathrm{Z}}_1& 0\\{}0& {\mathrm{Z}}_2\end{array}\right]\left[\begin{array}{@{}c@{}}{\mathrm{a}}_1\\{}{\mathrm{a}}_2\end{array}\right]+\left[\begin{array}{@{}c@{}}{\mathrm{e}}_1\\{}{\mathrm{e}}_2\end{array}\right]\kern1.25em \end{equation*}

$$ \left[\begin{array}{@{}c@{}}{\mathrm{a}}_1\\{}{\mathrm{a}}_2\end{array}\right]\sim N\left(0,\mathrm{M}\bigotimes \mathrm{G}\right),\left[\begin{array}{@{}c@{}}{\mathrm{e}}_1\\{}{\mathrm{e}}_2\end{array}\right]\sim N\left(0,\mathrm{R}\bigotimes \mathrm{I}\right) $$

where the subscripts 1 and 2 refer to populations 1 and 2, respectively, $\mathrm{y}$ is the vector of trait phenotypes (in population 1 or population 2), $\mathrm{b}$ is the vector of fixed effects, X is the design matrix of $\mathrm{b}$, which relates the effects in b to the phenotypes in $\mathrm{y}$; a is the vector of additive genetic effects of all individuals in both populations, Z is the design matrix of a to $\mathrm{y}$, which relates the effects in a to the phenotypes in $\mathrm{y}$; e is the vector of residual errors; $$\mathrm{M}=\left[\begin{array}{@{}cc@{}}{\sigma}_{{\mathrm{a}}_1}^2& {\sigma}_{{\mathrm{a}}_{12}}\\{}{\sigma}_{{\mathrm{a}}_{21}}& {\sigma}_{{\mathrm{a}}_2}^2\end{array}\right]$$ with ${\sigma}_{{\mathrm{a}}_1}^2$ and ${\sigma}_{{\mathrm{a}}_2}^2$ being the additive genetic variances and ${\sigma}_{{\mathrm{a}}_{12}}$ and ${\sigma}_{{\mathrm{a}}_{21}}$the additive genetic covariance for the two populations, $\mathrm{G}=\frac{{\mathrm{WW}}^{\prime }}{\sum 2{p}_j\left(1-{p}_j\right)}$ is the genomic relationship matrix (GRM) defined by VanRaden [21] with $\mathrm{W}$ being the centralized SNP genotype matrix with element *w_ij_* = *m_ij_* − 2*p_j_*, *m_ij_* being the genotype code (0, 1 and 2 for genotypes 11, 12 and 22, respectively) for individual *i* and SNP *j*, and ${p}_j$ being the minor allele frequency of SNP *j*; $$\mathrm{R}=\left[\begin{array}{@{}cc@{}}{\sigma}_{{\mathrm{e}}_1}^2& 0\\{}0& {\sigma}_{{\mathrm{e}}_2}^2\end{array}\right]$$ with ${\sigma}_{{\mathrm{e}}_1}^2$ and ${\sigma}_{{\mathrm{e}}_2}^2$ being the residual variances for the two populations.

The variance and covariance components involved in the model were estimated with residual maximum likelihood using the GCTA (v1.94.2) software [22]. Thus, the heritabilities of the trait in the two populations can be calculated as, ${h}_1^2=\frac{\sigma_{{\mathrm{a}}_1}^2}{\sigma_{{\mathrm{a}}_1}^2+{\sigma}_{{\mathrm{e}}_1}^2}$, ${h}_2^2=\frac{\sigma_{{\mathrm{a}}_2}^2}{\sigma_{{\mathrm{a}}_2}^2+{\sigma}_{{\mathrm{e}}_2}^2}$, and the genetic correlation can be calculated, ${r}_g=\frac{\sigma_{{\mathrm{a}}_{12}}}{\sqrt{\sigma_{{\mathrm{a}}_1}^2{\sigma}_{{\mathrm{a}}_2}^2}}$.

### Local genetic correlation estimation

Although the local genetic correlation can be estimated in the same way as the global genetic correlation with the G matrix being calculated with regional genomic variants, it is computationally infeasible for large datasets due to the large number of genomic regions. Alternatively, local genetic correlation can be approximately estimated using GWAS summary statistics [23] which are computational very efficient while maintain acceptable estimation accuracy. Several methods have been proposed, such as *ρ*-HESS [23], SUPERGNOVA [24], LOGODetect [25] and LAVA [26]. Here, we adapted the LAVA method because it provides unbiased estimation and well-controlled type-I error when using an in-sample reference panel according to a benchmark comparison by Zhang et al. [27]. We used the LAVA partitioning algorithm (v1.0.0) to divide the genome into independent LD blocks as described by Werme et al. [26] based on the combined genotype data of the two populations in each dataset. This algorithm recursively splitting the largest block into two smaller blocks, selecting a new breakpoint that minimizes local LD between the resulting blocks. Then, we performed single-trait GWAS based on imputed sequence data for each trait (i.e. population) to obtain the summary statistics data required for LAVA software (v0.1.0). Finally, the run.univ.bivar function of LAVA was run to estimate local genetic correlations. To ensure that LGC of all genomic regions were analyzed irrespective of whether the genetic signal of a region for the trait of interest is significant or not, we set the univariate *P* value significance threshold as 1 (univ.thresh = 1).

### GP models

For the trait of interest, which is phenotyped in two populations, the following models are used for genomic prediction.

#### STGBLUP

The STGBLUP (single-trait genomic best linear unbiased prediction) model is used for GP for the trait in each population separately. Here, the genetic correlations between populations are completely ignored. The model is as follows,


(2) \begin{equation*} \mathrm{y}=\mathrm{Xb}+\mathrm{Za}+\mathrm{e} \end{equation*}

$$ \mathrm{a}\sim N\left(0,\mathrm{G}{\sigma}_{\mathrm{a}}^2\right);\mathrm{e}\sim N\left(0,\mathrm{I}{\sigma}_{\mathrm{e}}^2\right) $$

where $\mathrm{y}$ is the vector of phenotypic values of the trait; $\mathrm{b}$ is the vector of fixed effects, $\mathrm{X}$ is the design matrix of $\mathrm{b}$, which relates the effects in b to the phenotypes in y; $\mathrm{a}$ is the vector of additive genetic effects, $\mathrm{Z}$ is the design matrix of a to y, which relates the effects in a to the phenotypes in y, ${\sigma}_{\mathrm{a}}^2$ is the addictive genetic variance and $\mathrm{G}$ is the GRM as defined in Model (1), $\mathrm{e}$ is the vector of the random residuals, and ${\sigma}_{\mathrm{e}}^2$ is the residual variance.

#### STGBLUP_combined

This model assumes that the trait in the two populations is defined (or measured) in the same way. The data of the two populations are combined to form a joint reference population. Then, GP for the trait is performed in the two populations respectively using STGBLUP and the joint reference population.

#### MTGBLUP

MTGBLUP is a multi-trait model in the GBLUP framework. Here, the same trait in the two populations are treated as two different traits and the global genetic correlation between them is considered in the analysis. The reference population is again a combination of the two populations, but one contributes phenotypes on one trait and the other contributes phenotypes on the other trait. GP is conducted for the two traits (i.e. populations) jointly. The model formula is the same as Model (1).

#### LGC-model-1

This is also a multiple trait model with the same trait in the two populations being treated as two different traits. But it employs the information of local (instead of global) genetic correlations (LGC) between populations for multi-trait genomic prediction. In this model, the genome is divided into two parts, the first part contains all regions with significant LGC (SIG regions) and the second part contains all rest regions (NON regions). Correspondingly, the vector $\mathrm{a}$ in Model (1) is also split into two sub-vectors and the model is as follows,


$$ \left[\begin{array}{@{}c@{}}{\mathrm{y}}_1\\{}{\mathrm{y}}_2\end{array}\right]=\left[\begin{array}{@{}cc@{}}{\mathrm{X}}_1& 0\\{}0& {\mathrm{X}}_2\end{array}\right]\left[\begin{array}{@{}c@{}}{\mathrm{b}}_1\\{}{\mathrm{b}}_2\end{array}\right]+ $$

(3) \begin{equation*} \left[\begin{array}{@{}cc@{}}{\mathrm{Z}}_{1_{\mathrm{SIG}}}& 0\\{}0& {\mathrm{Z}}_{2_{\mathrm{SIG}}}\end{array}\right]\left[\begin{array}{@{}c@{}}{\mathrm{a}}_{1_{\mathrm{SIG}}}\\{}{\mathrm{a}}_{2_{\mathrm{SIG}}}\end{array}\right]+\left[\begin{array}{@{}cc@{}}{\mathrm{Z}}_{1_{\mathrm{NON}}}& 0\\{}0& {\mathrm{Z}}_{2_{\mathrm{NON}}}\end{array}\right]\left[\begin{array}{@{}c@{}}{\mathrm{a}}_{1_{\mathrm{NON}}}\\{}{\mathrm{a}}_{2_{\mathrm{NON}}}\end{array}\right]+\left[\begin{array}{@{}c@{}}{\mathrm{e}}_1\\{}{\mathrm{e}}_2\end{array}\right] \end{equation*}

$$ \left[\begin{array}{@{}c@{}}{\mathrm{a}}_{1_{\mathrm{SIG}}}\\{}{\mathrm{a}}_{2_{\mathrm{SIG}}}\end{array}\right]\sim N\left(0,{\mathrm{M}}_{\mathrm{SIG}}\bigotimes{\mathrm{G}}_{\mathrm{SIG}}\right),\left[\begin{array}{@{}c@{}}{\mathrm{a}}_{1_{\mathrm{NON}}}\\{}{\mathrm{a}}_{2_{\mathrm{NON}}}\end{array}\right]\sim N\left(0,{\mathrm{M}}_{\mathrm{NON}}\bigotimes{\mathrm{G}}_{\mathrm{NON}}\right) $$

where ${\mathrm{a}}_{1_{\mathrm{SIG}}}$ and ${\mathrm{a}}_{2_{\mathrm{SIG}}}$ are the vectors of additive genetic effects owing to the SIG regions for the two traits, ${\mathrm{G}}_{\mathrm{SIG}}$ is the GRM constructed using SNPs in the SIG regions, $${\mathrm{M}}_{\mathrm{sig}}=\left[\begin{array}{@{}cc@{}}{\sigma}_{{\mathrm{a}}_{1_{\mathrm{SIG}}}}^2& {\sigma}_{{\mathrm{a}}_{12_{\mathrm{SIG}}}}\\{}{\sigma}_{{\mathrm{a}}_{21_{\mathrm{SIG}}}}& {\sigma}_{{\mathrm{a}}_{2_{\mathrm{SIG}}}}^2\end{array}\right]$$ is the additive genetic variance–covariance matrix of the two populations for the SIG regions; ${\mathrm{a}}_{1_{\mathrm{NON}}}$, ${\mathrm{a}}_{2_{\mathrm{NON}}}$, ${\mathrm{G}}_{\mathrm{NON}}$ and ${\mathrm{M}}_{\mathrm{NON}}$ are the counterparts of the above terms for the NON regions. The rest terms (${\mathrm{y}}_1$, ${\mathrm{y}}_2$, ${\mathrm{b}}_1$ and ${\mathrm{b}}_2$,) are the same as those in Model (1). In this model, total genomic estimated breeding values (GEBV) for two populations are defined as:


$$ {\mathrm{a}}_1={\mathrm{a}}_{1_{\mathrm{SIG}}}+{\mathrm{a}}_{1_{\mathrm{NON}}} $$

$$ {\mathrm{a}}_2={\mathrm{a}}_{2_{\mathrm{SIG}}}+{\mathrm{a}}_{2_{\mathrm{NON}}} $$

In this study, we defined the SIG regions as follows: we sorted the independent LD genome regions according to the *p*-values of their local genetic correlation estimates and the top 10 or top 20 regions are regarded as SIG regions.

#### LGC-model-2

This model is similar to LGC-model-1, but it divides the genome into three parts. The first part contains all regions with strong positive LGC (POS regions), the second contains all regions with strong negative LGC (NEG regions) and the third contains all rest regions (RES regions). The model is as follows,


$$ \left[\begin{array}{@{}c@{}}{\mathrm{y}}_1\\{}{\mathrm{y}}_2\end{array}\right]=\left[\begin{array}{@{}cc@{}}{\mathrm{X}}_1& 0\\{}0& {\mathrm{X}}_2\end{array}\right]\left[\begin{array}{@{}c@{}}{\mathrm{b}}_1\\{}{\mathrm{b}}_2\end{array}\right]+ $$

$$ \left[\begin{array}{@{}cc@{}}{\mathrm{Z}}_{1_{\mathrm{POS}}}& 0\\{}0& {\mathrm{Z}}_{2_{\mathrm{POS}}}\end{array}\right]\left[\begin{array}{@{}c@{}}{\mathrm{a}}_{1_{\mathrm{POS}}}\\{}{\mathrm{a}}_{2_{\mathrm{POS}}}\end{array}\right]+\left[\begin{array}{@{}cc@{}}{\mathrm{Z}}_{1_{\mathrm{NEG}}}& 0\\{}0& {\mathrm{Z}}_{2_{\mathrm{NEG}}}\end{array}\right]\left[\begin{array}{@{}c@{}}{\mathrm{a}}_{1_{\mathrm{NEG}}}\\{}{\mathrm{a}}_{2_{\mathrm{NEG}}}\end{array}\right]+ $$

(4) \begin{equation*} \left[\begin{array}{@{}cc@{}}{\mathrm{Z}}_{1_{\mathrm{RES}}}& 0\\{}0& {\mathrm{Z}}_{2_{\mathrm{RES}}}\end{array}\right]\left[\begin{array}{@{}c@{}}{\mathrm{a}}_{1_{\mathrm{RES}}}\\{}{\mathrm{a}}_{2_{\mathrm{RES}}}\end{array}\right]+\left[\begin{array}{@{}c@{}}{\mathrm{e}}_1\\{}{\mathrm{e}}_2\end{array}\right] \end{equation*}

$$ \left[\begin{array}{@{}c@{}}{\mathrm{a}}_{1_{\mathrm{POS}}}\\{}{\mathrm{a}}_{2_{\mathrm{POS}}}\end{array}\right]\sim N\left(0,{\mathrm{M}}_{\mathrm{POS}}\bigotimes{\mathrm{G}}_{\mathrm{POS}}\right),{\mathrm{M}}_{\mathrm{POS}}=\left[\begin{array}{@{}cc@{}}{\sigma}_{{\mathrm{a}}_{1_{\mathrm{POS}}}}^2& {\sigma}_{{\mathrm{a}}_{12_{\mathrm{POS}}}}\\{}{\sigma}_{{\mathrm{a}}_{21_{\mathrm{POS}}}}& {\sigma}_{{\mathrm{a}}_{2_{\mathrm{POS}}}}^2\end{array}\right] $$

$$ \left[\begin{array}{@{}c@{}}{\mathrm{a}}_{1_{\mathrm{NEG}}}\\{}{\mathrm{a}}_{2_{\mathrm{NEG}}}\end{array}\right]\sim N\left(0,{\mathrm{M}}_{\mathrm{NEG}}\bigotimes{\mathrm{G}}_{\mathrm{NEG}}\right),{\mathrm{M}}_{\mathrm{NEG}}=\left[\begin{array}{@{}cc@{}}{\sigma}_{{\mathrm{a}}_{1_{\mathrm{NEG}}}}^2& {\sigma}_{{\mathrm{a}}_{12_{\mathrm{NEG}}}}\\{}{\sigma}_{{\mathrm{a}}_{21_{\mathrm{NEG}}}}& {\sigma}_{{\mathrm{a}}_{2_{\mathrm{NEG}}}}^2\end{array}\right] $$

$$ \left[\begin{array}{@{}c@{}}{\mathrm{a}}_{1_{\mathrm{RES}}}\\{}{\mathrm{a}}_{2_{\mathrm{RES}}}\end{array}\right]\sim N\left(0,{\mathrm{M}}_{\mathrm{RES}}\bigotimes{\mathrm{G}}_{\mathrm{RES}}\right),{\mathrm{M}}_{\mathrm{RES}}=\left[\begin{array}{@{}cc@{}}{\sigma}_{{\mathrm{a}}_{1_{\mathrm{RES}}}}^2& {\sigma}_{{\mathrm{a}}_{12_{\mathrm{RES}}}}\\{}{\sigma}_{{\mathrm{a}}_{21_{\mathrm{RES}}}}& {\sigma}_{{\mathrm{a}}_{2_{\mathrm{RES}}}}^2\end{array}\right] $$

All the terms in the model are the same as those in Model (1) with the subscripts POS, NEG and RES refer to the corresponding genomic regions. In this model, the total GEBV for two populations are defined as:


$$ {\mathrm{a}}_1={\mathrm{a}}_{1_{\mathrm{POS}}}+{\mathrm{a}}_{1_{\mathrm{NEG}}}+{\mathrm{a}}_{1_{\mathrm{RES}}} $$

$$ {\mathrm{a}}_2={\mathrm{a}}_{2_{\mathrm{POS}}}+{\mathrm{a}}_{2_{\mathrm{NEG}}}+{\mathrm{a}}_{2_{\mathrm{RES}}} $$

In this study, we defined a region as POS (NEG) region if $\mid{\hat{r}}_{lgc}\mid$ ≥ 0.5 (or 0.6), where ${\hat{r}}_{lgc}$ is the estimated local genetic correlation (*lgc*).

### Evaluation of the performance of the proposed models

We used two real datasets to evaluate the performances of the proposed models. A summary of these datasets is shown in Table 1.

**Table 1:** Summary of the mice and cow datasets used in this study

Datasets	Population	Phenotypes^a^	No. individuals
Mice	OX	startle	1478
		fc.cue.corr	1939
		bmd.a	1936
		TA	1908
		soleus	1911
	UC	startle	815
		fc.cue.corr	1037
		bmd.a	1037
		TA	1013
		soleus	1027
Cow	CN	FY	6649
		PY	
		MY	
		FP	
		PP	
	SD	FY	1677
		PY	
		MY	
		FP	
		PP	

^a^fc.cue.corr: corrected freezing to cue, bmd.a: abnormal bone mineral density, TA: weight of tibialis anterior, soleus: weight of soleus, FY: milk fat yield, PY: milk protein yield, MY: milk yield, FP: milk fat percentage, PP: milk protein percentage.

#### Mice dataset

We leveraged two outbred mice (the Crl:CFW(SW)-US_P08 stock) populations, one from the University of Oxford (OX) in the United Kingdom [28] and the other from the University of Chicago (UC) in the United States of America [29]. The OX mice were genotyped by low coverage sequencing, and the UC mice were genotyped by GBS (genotype by sequencing). Zou et al. [30] combined the two populations together and carried out imputation using STITCH [31]. All phenotypes had been corrected for systematical environmental effects, including sex, weight and batch, through a linear model, followed by quantile normalization. Therefore, there were no fixed effects except the overall mean, i.e. vector $\mathrm{b}$ in all models contained the overall mean only. We employed the phenotypic and genotypic datasets as curated by Zou et al. [30]. Five traits including startle, corrected freezing to cue (fc.cue.corr), abnormal bone mineral density (bmd.a), weight of tibialis anterior (TA) and weight of soleus (soleus) were considered in this study. The dataset comprised 1940 mice from the OX population and 1037 from the UC population. We removed SNPs with call rate less than 0.01, minor allele frequency (MAF) lower than 0.05 or *p*-value <1.0E-6 for Hardy–Weinberg Equilibrium test, resulting in 2,058,668 SNPs for subsequent analyses.

#### Cow dataset

Two Chinese Holstein populations were used in this study, the Shandong population (SD) which contains 1677 cows from the Shandong Province of China and the national population (CN) which contains 6649 cows from other parts of China. The cows of the CN population were genotyped using various types of SNP chips, including Illumina Bovine SNP50v1 (50 K), Illumina Bovine SNP50v2 (50 K) and GeneSeek Genomic Profiler Bovine HD (80 K), while the cows of the SD population were genotyped exclusively with the GeneSeek Genomic Profiler Bovine 100 K chip. All chip data was imputed to whole-genome sequence (WGS) level using Beagle5 [32], as elaborated in our prior study [33]. SNPs with MAF less than 0.05 or with *p*-value <1.0E-6 for Hardy–Weinberg Equilibrium test were excluded, resulting in 11,133,463 SNPs for subsequent analysis. Five milk production traits including milk fat yield (FY), milk protein yield (PY), milk yield (MY), milk fat percentage (FP) and milk protein percentage (PP) were analyzed. De-regressed proofs (DRP) derived from estimated breeding values (EBV) using the method of Garrick et al. [34] were used as pseudo phenotype in the subsequent analysis. Therefore, there were no fixed effects and vector b in all models contained the overall mean only. The DRPs for the CN population were derived from the official EBVs from Dairy Association of China. These EBVs were obtained based on a multi-trait multi-lactation random regression test-day model. The DRPs for the SD population were stemmed from the official EBVs from the Shandong Dairy Cattle Center, which were obtained based on a single-trait single-lactation random regression test-day model. Therefore, the DRPs in the two populations were different in nature so that the model STGBLUP_combined is not applicable for this dataset.

We used a 10-fold cross-validation (CV) to assess the accuracy of the genomic prediction. The dataset was randomly split into 10 subsets of equal size and one of them in turn was used as validation population and the rest nine were used as training population. To obtain stable results, we repeat the 10-fold CV for 10 times. For the mice dataset, the accuracy of GP for the validation individuals was assessed by ${r}_{y_c, GEBV}$, correlation between corrected phenotypic values (${y}_c$) and GEBV and the unbiasedness of prediction was assessed by${b}_{y_c, GEBV}$, the regression of ${y}_c$ on GEBV. For the cow dataset, the accuracy was assessed by ${r}_{DRP, GEBV}$, correlation between DRP and GEBV of the validation individuals and the unbiasedness of predictions was assessed by${b}_{DRP, GEBV}$.

## Results

### Population structure and genetic parameters

To evaluate the population structure of the datasets, we conducted principal component analysis using SNP genotypes. For the mice dataset, Figure S1A shows that the genetic backgrounds of the OX and UC populations are similar, but there are some stratifications within population. However, for the cow dataset, Figure S1B shows that some individuals in the CN population have quite different genetic background from the SD population.

**Figure 1:**
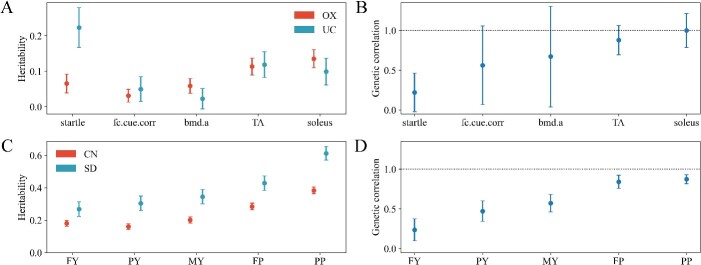
Estimates of heritabilities and between-population genetic correlations of the traits in mice and cow datasets. (A) Heritabilities for the five traits in the OX and UC populations of mice. (B) Global genetic correlations between the OX and UC populations of mice for the five traits. (C) Heritabilities for the five traits in the CN and SD populations of cows. (D) Global genetic correlations between the CN and SD populations of cows for the five traits. The dots represent the estimated heritabilities or genetic correlations, and the error bars show the standard errors of the estimates. Fc.Cue.Corr: Corrected freezing to cue. Bmd.A: Abnormal bone mineral density. TA: Weight of TA. Soleus: Weight of soleus. FY: Milk fat yield. PY: Milk protein yield. MY: Milk yield. FP: Milk fat percentage. PP: Milk protein percentage.

**Figure 2:**
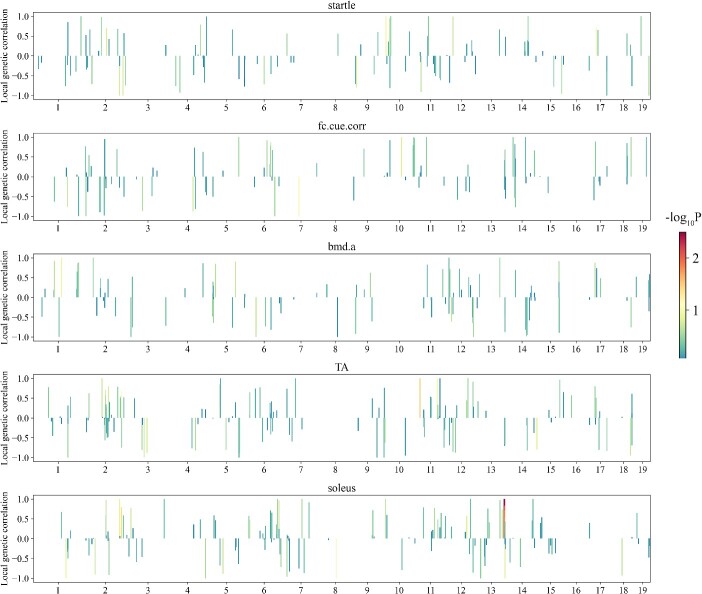
Estimates of between-population local genetic correlations of the five traits in the mice dataset. The color of each bar indicates the significance status measured with -log_10_P of the estimates. Fc.Cue.Corr: Corrected freezing to cue. Bmd.A: Abnormal bone mineral density. TA: Weight of TA. Soleus: Weight of soleus.

We estimated the heritabilities and global between-population genetic correlations for all traits using model (1). The results are shown in Fig. 1. For the mice dataset, all the five traits exhibited low heritability in both populations, all estimated heritabilities are less than 0.15 except the trait startle in the UC population which had an estimated heritability higher than 0.2 (Fig. 1A). The estimated global genetic correlations for the five traits between the OX and UC populations ranged from 0.2205 for startle to 1.0 for soleus (Fig. 2B). For the cow dataset, the SD population exhibited higher heritability than the CN population for all five traits (Fig. 1C). The estimated global genetic correlations between the CN and SD populations for the five traits ranged from 0.2364 for FY to 0.8399 and 0.8729 for FP and PP. It is worth noting that the standard errors of the estimates for the mice dataset were larger than that for the cow dataset. In particular, the standard errors of the estimated genetic correlations for the mice data were very large.

### Between-population local genetic correlations

**Figure 3:**
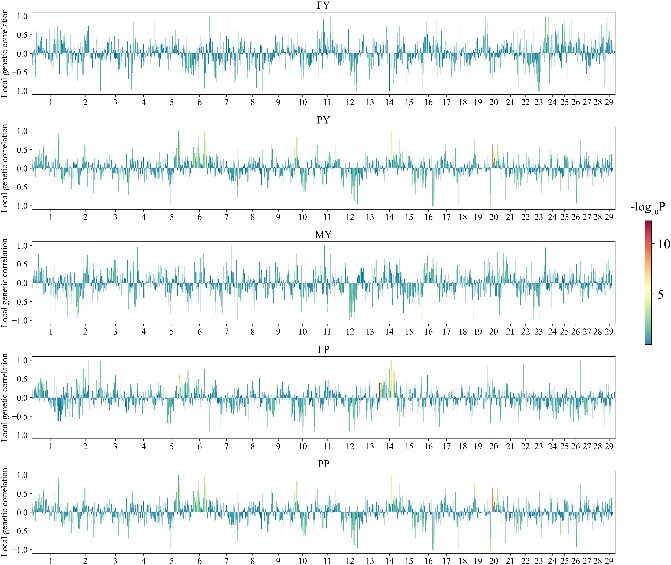
Estimates of between-population local genetic correlations for the five traits in the cow datasets. The color of each bar indicates the significance status measured with -log_10_P of the estimates. FY: Milk fat yield. PY: Milk protein yield. MY: Milk yield. FP: Milk fat percentage. PP: Milk protein percentage.

Using the LAVA partitioning algorithm, we portioned the mouse genome into 696 regions (independent LD blocks) with an average size of 3.23 Mb and the cow genome into 3249 regions with an average size of 0.77 Mb (Figure S2). The large difference in LD block sizes between the two datasets are due to the much higher density of SNPs used in the cow population (11 133 463 SNPs) than that in the mice population (2 058 668 SNPs), as well as the much higher LD level in the mice population than that in the cow population, as shown in Figure S1C. The between-population local genetic correlation estimates for all traits of the mice and cow datasets are visually presented in Figs 2 and 3, respectively. Notably, the between-population LGC in different regions show large variations across the genome. Specifically, for all traits, some regions exhibit strong positive correlations, some exhibit strong negative correlations and some exhibit notably low or nearly zero correlations. For some traits, while the global genomic correlations are highly positive, there are regions which exhibited weak correlations or strong negative correlations, such as the trait soleus of mice (Fig. 2) and the trait PP of cow (Fig. 3). These observations reveal the complex nature of genetic correlations.

### GP using single trait and multi-trait models

**Figure 4:**
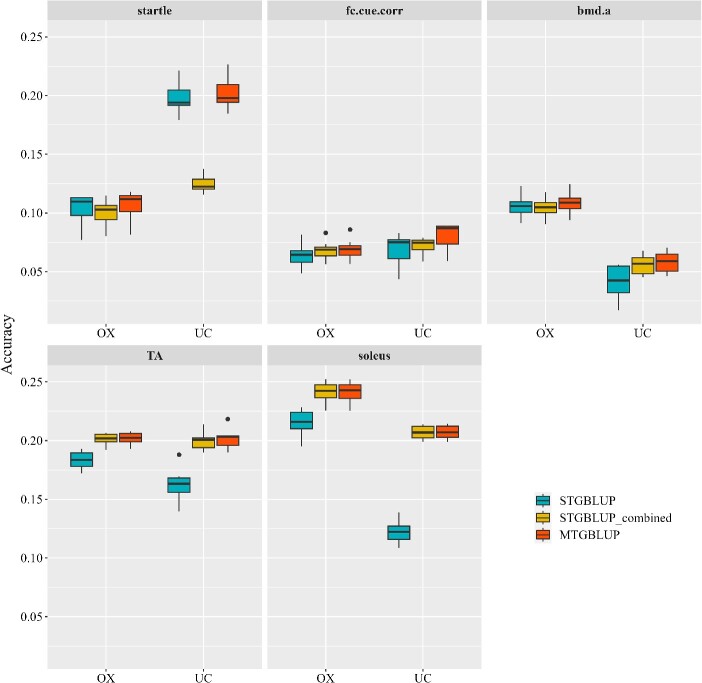
Genomic prediction accuracies of the single trait model (STGBLUP and STGBLUP_combined) and the conventional multi-trait model (MTGBLUP) in the mice dataset. Fc.Cue.Corr: Corrected freezing to cue. Bmd.A: Abnormal bone mineral density. TA: Weight of TA. Soleus: Weight of soleus.

We compared the prediction performances using the single trait models, STGBLUP and STGBLUP_combined and the multi-trait model, MTGBLUP. The two single trait models conduct GP for each trait in each population separately. They differ in that STGBLUP uses single population as reference population, while STGBLUP_combined uses the combined population as reference population. Both models ignore the between-population genetic correlation. MTGBLUP regards the same trait in two populations as two different traits and conducts GP for the two traits jointly by considering the global between-population genetic correlation. Fig. 4 shows the prediction accuracies of these models for the five traits in the mice dataset. STGBLUP performed the worst in all cases, except for the trait startle, for which STGBLUP_combined performed the worst. This should be due to the low between-population genetic correlation for this trait (${\hat{r}}_g=0.2205$), indicating large G × E interaction, such that it is unreasonable to treat the trait in the two populations as the same trait. STGBLUP_combined and MTGBLUP performed equally except for the trait startle. Their superiorities over STGBLUP increased with the increase of the between-population genetic correlations. In particular, for traits TA and soleus, which exhibited between-population correlations of 0.8784 and 1.0, respectively, their relative superiorities reached 9.97–69.70%. In addition, the GEBVs from MTGBLUP were less biased compared to those from STGBLUP, as shown in Figure S3.

**Figure 5:**
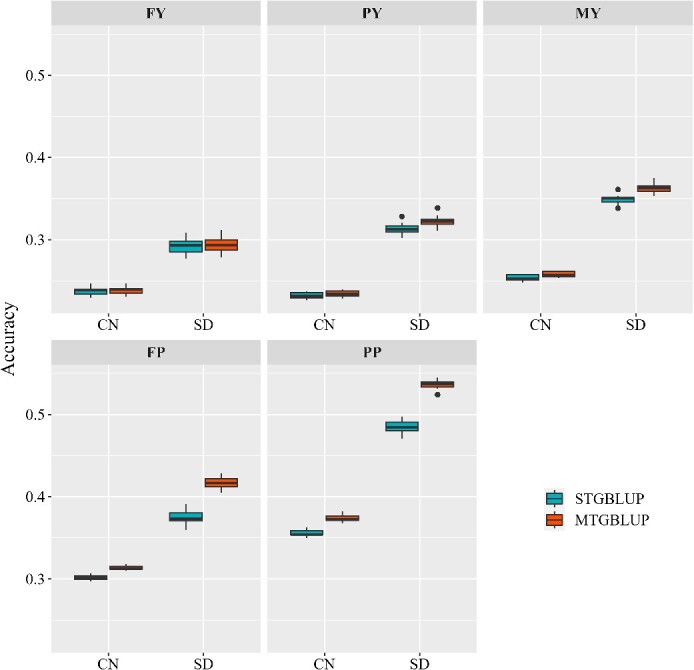
Genomic prediction accuracies of STGBLUP and MTGBLUP in the CN and SD populations of the cow dataset. FY: Milk fat yield. PY: Milk protein yield. MY: Milk yield. FP: Milk fat percentage. PP: Milk protein percentage.

In the analysis of the cow dataset, only STGBLUP and MTGBLUP were compared since the STGBLUP_combined model is not applicable to this dataset. For these two models, similar results (Fig. 5) were obtained to those for the mice dataset. MTGBLUP outperformed STGBLUP for all traits in both populations, and the higher the between-population genetic correlation, the larger superiority of MTGBLUP over STGBLUP. For FP and PP, which showed very high between-population genetic correlations (over 0.8), the relative superiorities of MTGBLUP reached 4.03–11.28%. There was no obvious improvement of the unbiasedness of GEBVs from MTGBLUP compared to that from STGBLUP (Figure S4).

### GP using models integrating local genetic correlations

We compared the MTGBLUP model and the two LGC models proposed in this study for multi-population GP using the mice and cow datasets.

#### Mice dataset

**Figure 6:**
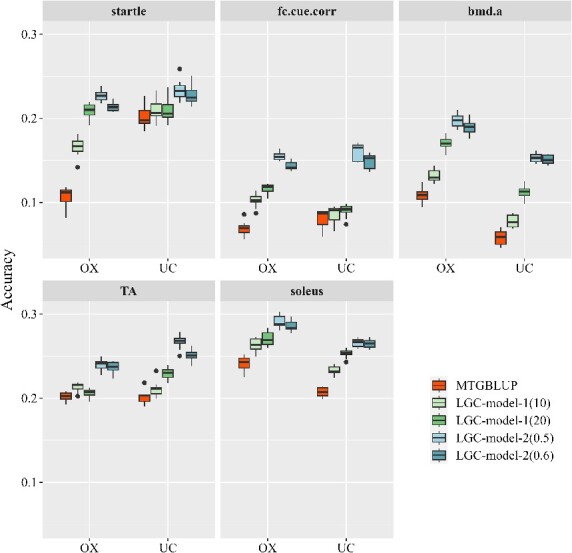
Genomic prediction accuracies of MTGBLUP, LGC-model-1, and LGC-model-2 in the OX and UC populations of the mice dataset. For LGC-model-1, two thresholds for significance were applied: Top 10 regions (i.e. the 10 most significant regions) and top 20 regions. For LGC-model-2, two thresholds for distinguishing strong correlations were applied: $\left|{\hat{r}}_{lgc}\right|$ ≥ 0.5 and $\left|{\hat{r}}_{lgc}\right|$ ≥ 0.6. Fc.Cue.Corr: Corrected freezing to cue. Bmd.A: Abnormal bone mineral density. TA: Weight of tibialis anterior. Soleus: Weight of soleus.

Fig. 6 shows the prediction accuracies of the three models with different thresholds for the LGC models, i.e. significant regions = top 10 or 20 for LGC-model-1, and strong LGC when $\left|{\hat{r}}_{lgc}\right|$ ≥ 0.5 or 0.6 for LGC-model-2. The results indicate that the two LGC models outperformed MTGBLUP in all cases. The relative superiority of LGC-model-1 over MTGBLUP ranged from 1.93% to 97.26%, and the relative superiority of LGC-model-2 over MTGBLUP ranged from 13.08% to 163.86%. The choice of the thresholds for LGC-model-1 and LGC-model-2 had significant effects on their performance. Specifically, LGC-model-1 with a threshold of top 20 generally outperformed that with a threshold of top 10. For LGC-model-2, the threshold of $\left|{\hat{r}}_{lgc}\right|$ ≥ 0.5 yielded better performance than $\left|{\hat{r}}_{lgc}\right|$ ≥ 0.6. Moreover, LGC-model-2 performed better than LGC-model-1 for all traits in both populations.

The unbiasedness of the three models are shown in Figure S5. For all the five traits, both LGC-model-1 and LGC-model-2 exhibit smaller biases in comparison to MTGBLUP.

#### Cow dataset

**Figure 7:**
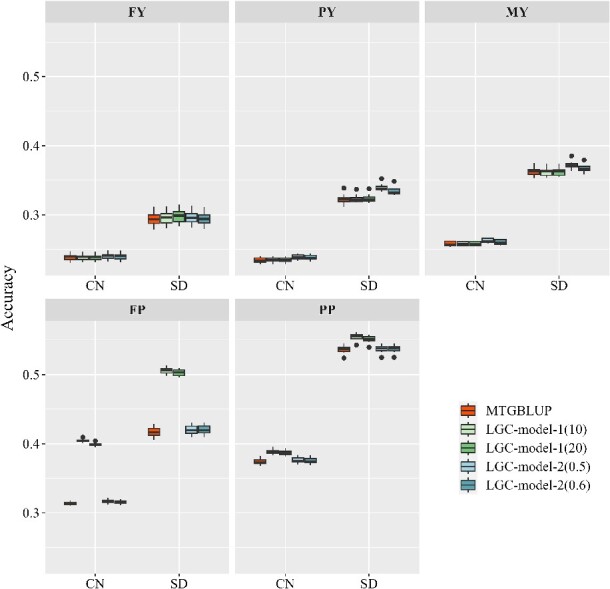
Genomic prediction accuracies of MTGBLUP, LGC-model-1 and LGC-model-2 in the CN and SD populations of the cow dataset. For LGC-model-1, two thresholds for significance were applied: Top 10 regions (i.e. the 10 most significant regions) and top 20 regions. For LGC-model-2, two thresholds for distinguishing strong correlations were applied: $\left|{\hat{r}}_{lgc}\right|$ ≥ 0.5 and $\left|{\hat{r}}_{lgc}\right|$ ≥ 0.6. FY: Milk fat yield. PY: Milk protein yield. MY: Milk yield. FP: Milk fat PERCENTAGE. PP: Milk protein percentage.

For the cow dataset, the two LGC models performed equally to or better than MTGBLUP (Fig. 7) in all cases. LGC-model-1 performed the best for traits FP and PP. For trait FP, it increased the prediction accuracy over MTGBLUP by 28.10% and 20.88% in population CN and SD, respectively. For trait PP, the improvements were 3.64% and 3.08%, respectively. For the other three traits, it produced the same accuracies as MTGBLUP. Different thresholds (top 10 or top 20) did not differ much. LGC-model-2 performed the best for traits PY and MY, but the superiorities in accuracy were rather small (0.84–5.20%). For the other three traits, it performed the same as MTGBLUP.

The unbiasedness of GEBVs from the three models did not differ significantly for all traits, as shown in Figure S6.

## Discussion

Setting up a joint reference population by combining different populations is a common approach for multi-population GP [35–37]. However, there are two requirements underlying this approach, first, the trait of interest must be defined or measured in the same way in different populations, and second, there is no G × E interaction in different populations, or equivalently, the performances of the trait expressed in different populations (environments) can be regarded as the same trait. However, in practice these requirements are often not met. One way to overcome this problem is to treat performances of the trait in different populations as different traits and perform GP using a multi-trait model by considering the between-population genetic correlation. Under this model, it is neither necessary to define or measure the trait in different populations in the same way, nor to assume no G × E interaction. In this study, we compared the models STGBLUP, STGBLUP_combined and MTGBLUP. STGBLUP simply performs GP for the trait of interest in each population separately using single trait GBLUP. STGBLUP_combined regards the trait of interest in different population as the same trait and combines different populations to form a joint reference population, and then performs GP in each population using single trait GBLUP. MTGBLUP treats the trait in different populations as different traits and performs multi-population GP using multi-trait GBLUP by considering the between-population genetic correlation. Using two real datasets, a mice dataset and a cow dataset, both containing data from two populations with varying between-population genetic correlations for different traits, we demonstrated that STGBLUP_combined and MTGBLUP could indeed improve the prediction accuracy compared to STGBLUP, particularly for traits with high between-population genetic correlation. STGBLUP_combined and MTGBLUP performed equally in most cases. However, for traits with low between-population genetic correlation (indicating strong G × E interaction), STGBLUP_combined performed worse than STGBLUP, while MTGBLUP still slightly better than STGBLUP, indicating that STGBLUP_combined is not suitable for traits with strong G × E interaction, as mentioned before, while MTGBLUP is robust to G × E interaction. It should be noted that, in the cow dataset, the CN population is much larger than the SD population, but the prediction accuracies in the CN population were lower than that in the SD population (Fig. 5). There are two important differences between the two populations. First, the SD population exhibited higher heritabilities than the CN population for all traits (Fig. 1C). Second, the individuals in the SD population are more closely grouped together than those in the CN population (Figure S1B). Thus, in the cross validation to assess the accuracy of the genomic prediction, the training and validation individuals for the SD population had closer relationship than those in the CN population. Both of these two aspects benefit the accuracy of GP in the SD population.

Although MTGBLUP is proved to be a good model for multi-population genomic prediction, it can be further improved by incorporating between-population LGCs. We demonstrated that LGCs varied greatly across the genome. For any trait, no matter how large the between-population global genetic correlation is, there are regions with strong positive, regions with strong negative and regions with weak or even zero LGCs. The global genetic correlation is the integration of all LGCs. Therefore, it is expected a model that employs LGC information for multi-trait GP should be more efficient than a model employing global genetic correlation information, such as MTGBLUP. In this study, we proposed two LGC models, LGC-model-1 and LGC-model-2, for multi-population genomic prediction. They are basically also multi-trait GBLUP model, but incorporate LGC information into the model by classifying the genomic regions into different classes according to the LGC information associated with these regions. LGC-model-1 considers the significance of LGCs and partitions the genome regions into two classes, i.e. regions with significant LGCs (with certain threshold) and regions with non-significant LGCs. LGC-model-2 considers the size and direction of LGCs and partitions the genome into three classes, i.e. regions with strong positive LGCs (with certain threshold), regions with strong negative LGCs and the rest regions (with weak or zero LGCs). We compared the two models with MTGBLUP using the two real datasets. For the mice dataset, both LGC models outperformed MTGBLUP for all five traits with the relative improvements ranging from 1.93% to 163.86%. LGC-model-2 performed consistently better than LGC-model-1. For the cow dataset, the two models performed better than or equally well as MTGBLUP, and the maximum relative improvement reached 28.97%. LGC-model-1 performed better in for traits FP and PP. The different relative superiority between the two models in the two datasets and for different traits can be explained as follows. Since LGC-model-1 partitions the genome based on the significance levels of LGCs, it is expected that it would have good performance in scenarios where there are some regions which contain SNPs with large effects on both traits and thus exhibit highly significant LGCs. This is the case in the cow dataset. In dairy cattle, it is well-known that the *DGAT1* gene is a major gene for FP and the *GHR* gene is a major gene for PP [38]. In our study, we found that the LGCs in the *DGAT1* region (Chr14:0–1.02 Mb) and the *GHR* region (Chr20: 30.2–31.7 Mb) were extremely significant with *P* values of 5.54E-13 and 1.25E-9, respectively. However, there are no such regions in the mouse genome. Therefore, LGC-model-1 did not show advantage over LGC-model-2 in the mice dataset.

An issue related to the LGC models is that they need to define the thresholds for classifying the genome regions, i.e. regions with or without significant LGCs (for LGC-model-1) and regions with strong or weak LGCs (LGC-model-2). In this study, for LGC-model-1, we defined a LGC to be significant if its *p-*value (−log(*p*)) was within the top 10 or top 20, for LGC-model-2, we defined a LGC to be strong positive or negative if $\left\lfloor{\hat{\boldsymbol{r}}}_{\boldsymbol{lgc}}\right\rfloor \ge \mathbf{0.5}\ \mathbf{or}\ \mathbf{0.6}$. We found that different thresholds for the two models had some effects on their performance, but there was no general trend that showed which threshold was better universally. For LGC-model-1, the threshold of top 20 yielded consistently better prediction accuracy than the threshold of top 10 for the mice dataset, while for the cow dataset, the two thresholds did not make significant difference. For LGC-model-2, the threshold of $\left\lfloor{\hat{\boldsymbol{r}}}_{\boldsymbol{lgc}}\right\rfloor \ge \mathbf{0.5}$ resulted in slightly better accuracy than the threshold of $\left\lfloor{\hat{\boldsymbol{r}}}_{\boldsymbol{lgc}}\right\rfloor \ge \mathbf{0.6}$ for the mice dataset, while for the cow dataset, again the two thresholds did not make significant difference. In view of these results, it might be a good choice to use top 20 as a generic threshold for LGC-model-1 and $\left\lfloor{\hat{\boldsymbol{r}}}_{\boldsymbol{lgc}}\right\rfloor \ge \mathbf{0.5}$ as a generic threshold for LGC-model-2 because the LGC models with these thresholds performed better than or equally to those with the alternative thresholds in most cases.

## Conclusions

In this study, we demonstrated that MTGBLUP (multi-trait genomic BLUP) which employs global between-population genetic correlation is an efficient model for multi-population GP and outperforms the conventional multi-population model which simply combines different populations together. We further proposed that MTGBLUP can be improved by partitioning the global between-population genetic correlation into LGC. We suggested two LGC models, LGC-model-1 and LGC-model-2, which partition the genome into regions with and without significant LGC (LGC-model-1) or regions with and without strong LGC (LGC-model-2). Using real datasets, we demonstrated that the LGC models could increase universally prediction accuracy and their relative improvement over MTGBLUP reached up to 163.86% (25.64% on average).

**Key Points**

- The between-population LGC in different regions show large variations across the genome.
- MTGBLUP outperformed the conventional multi-population model which simply combines different populations together (STGBLUP_combined).
- LGC models could increase universally prediction accuracy and their relative improvement over MTGBLUP reached up to 163.86%.

## Supplementary Material

Supplementary_file_bbae276

## Data Availability

The genotype and phenotype data for mice are available at https://doi.org/10.1093/g3journal/jkab394. The genotype and phenotype data for dairy cow used in the current study are available from the corresponding authors upon reasonable request. The code for running the LGC models along with an example dataset can be found at https://github.com/Tengjun0520/LGC-model_for_multi-population_genomic_prediction.

## Author Biographies

**Jun Teng** is a postdoctoral researcher at Shandong Agricultural University. He is currently engaged in genome-wide association studies and genomic prediction.

**Tingting Zhai** is a postdoctoral researcher at Shandong Agricultural University. She is currently focused on the regulation and function of plant small RNA molecular synthesis and metabolism.

**Xinyi Zhang** is a PhD student in animal breeding and genetics at Shandong Agricultural University. She is currently working on transcriptome-wide association studies.

**Changheng Zhao** is a PhD student in animal breeding and genetics at Shandong Agricultural University. He is currently researching breed identification.

**Wenwen Wang** is a professor at Shandong Agricultural University. She specializes in the mining and validation of functional genes for economically important traits in livestock and poultry.

**Hui Tang** is a professor at Shandong Agricultural University. He has worked in quantitative genetics and statistical genomics.

**Dan Wang** is a professor at Shandong Agricultural University. She focuses on statistical genomics and the evolution and domestication of native animals.

**Yingli Shang** is a professor at Shandong Agricultural University. He is dedicated to the prevention and control of zoonotic diseases.

**ChaoNing** is a professor at Shandong Agricultural University. He is currently engaged in computational biology and bioinformatics, including genome-wide association study and omics analysis.

**Qin Zhang** is a professor at Shandong Agricultural University. He has extensive research in various fields of bioinformatics and computational biology, including quantitative genetics and statistical genomics.
