# Supplementary material for: Improving multi-population genomic prediction accuracy using multi-trait GBLUP models which incorporate global or local genetic correlation information
Source: Brief Bioinform. 2024 Jun 10;25(4):bbae276. doi: 10.1093/bib/bbae276 (PMC11163384; doi:10.1093/bib/bbae276)
Supplement: Supplementary_file_bbae276 [file supplementary_file_bbae276.docx]

**
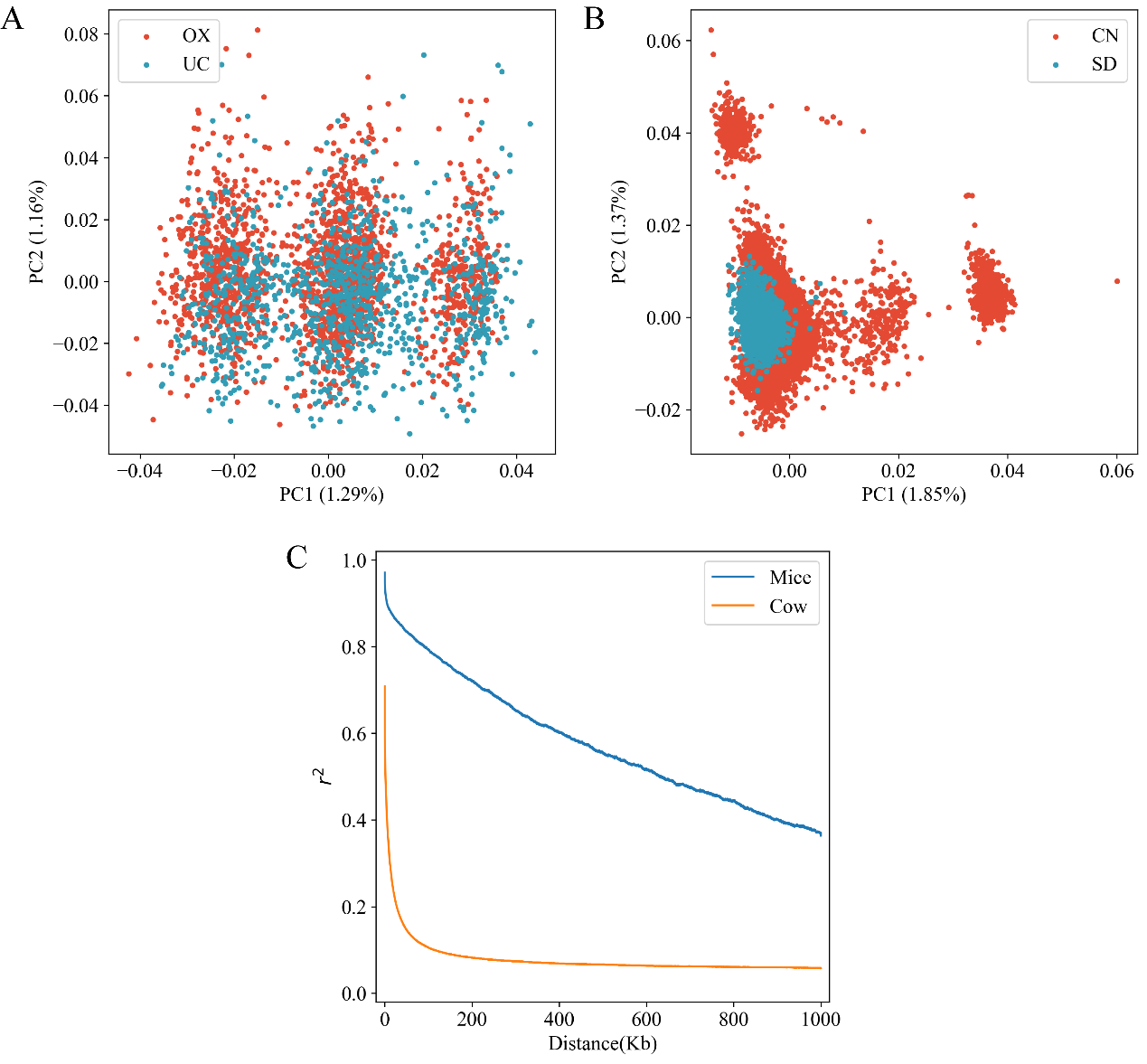
**

**Figure S1.** Population structure analysis of the mice and cow datasets. Principal component analysis (PCA) for populations in the mice (A) and cow (B) datasets. PC1: first principal component; PC2: second principal component. (C) The extent of linkage disequilibrium (LD) in mice and cow. Values are mean LD *r*^2^ values for all pairs of SNPs binned by distance.


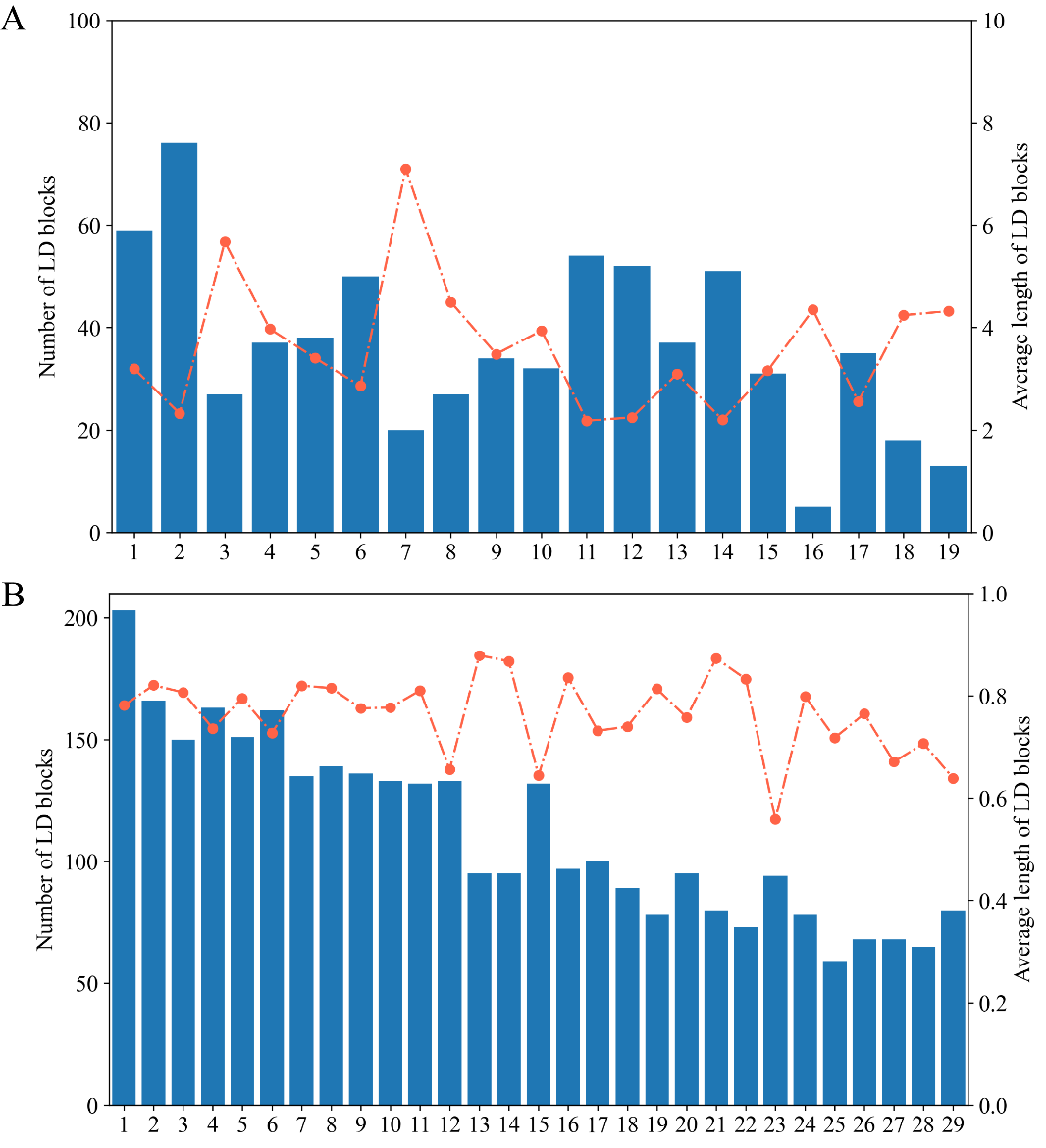


**Figure S2.** Numbers and average lengths of the independent linkage disequilibrium (LD) blocks determined by the LAVA partitioning algorithm on each chromosomes of the mouse (A) and cow (B) genome. The blue bars represent the numbers of LD blocks, the orange lines indicate the average lengths of the LD blocks.


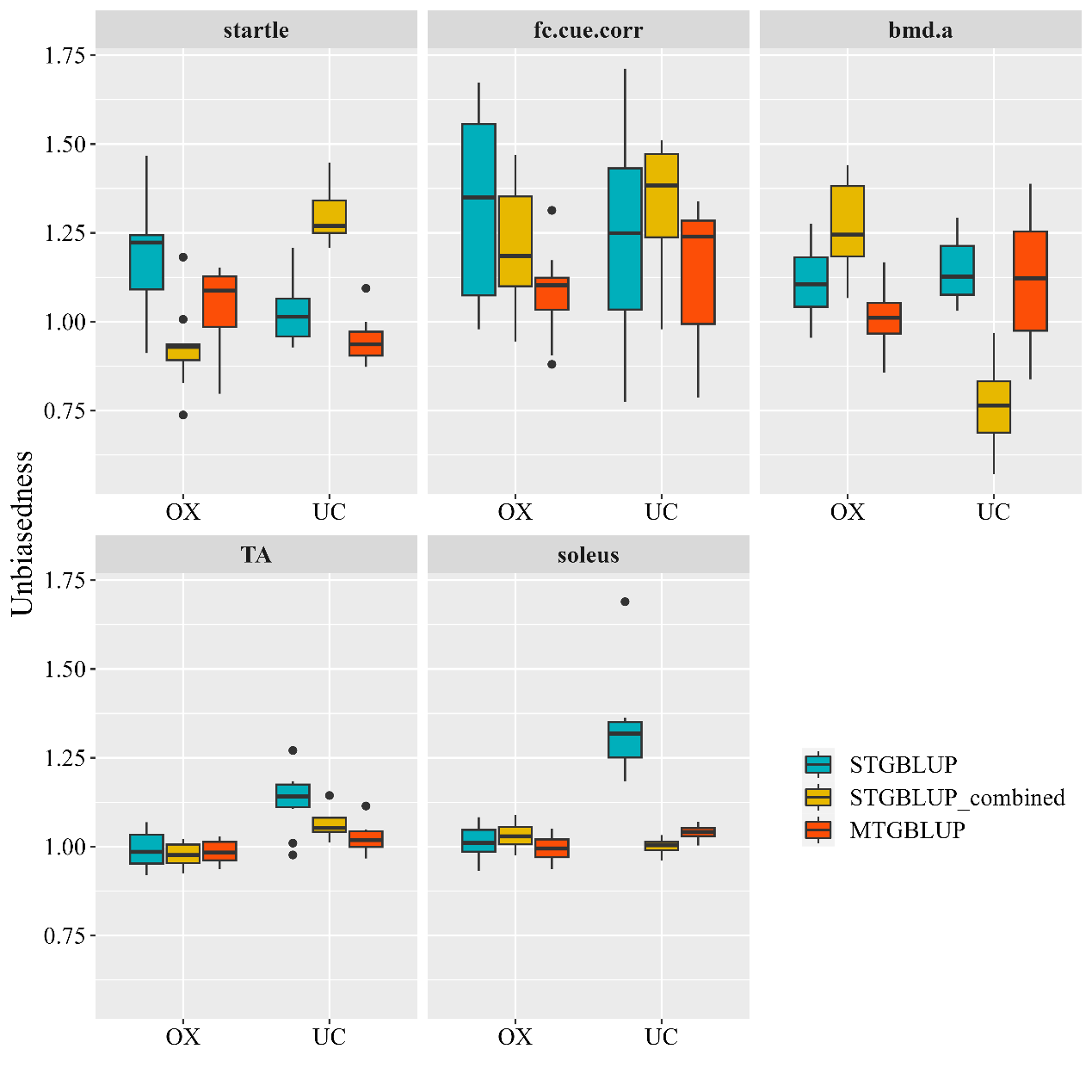


**Figure S3.** Unbiasedness of genomic prediction of STGBLUP, STGBLUP_combined, and MTGBLUP in the OX and UC populations of the mice dataset. Fc.cue.corr: corrected freezing to cue. Bmd.a: abnormal bone mineral density. TA: weight of tibialis anterior. Soleus: weight of soleus.


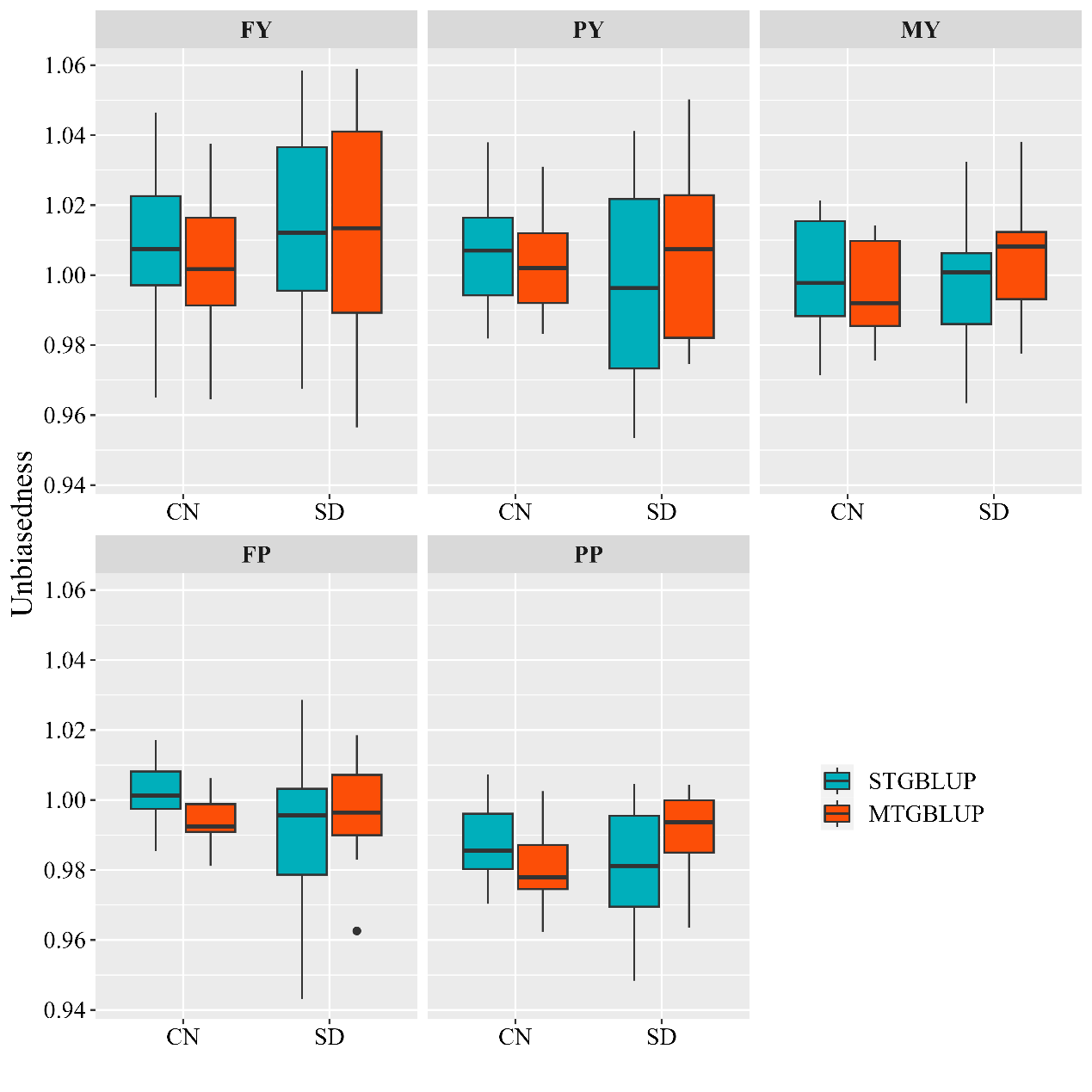


**Figure S4.** Unbiasedness of genomic prediction of STGBLUP and MTGBLUP in the CN and SD populations of the cow dataset. FY: milk fat yield. PY: milk protein yield. MY: milk yield. FP: milk fat percentage. PP: milk protein percentage.


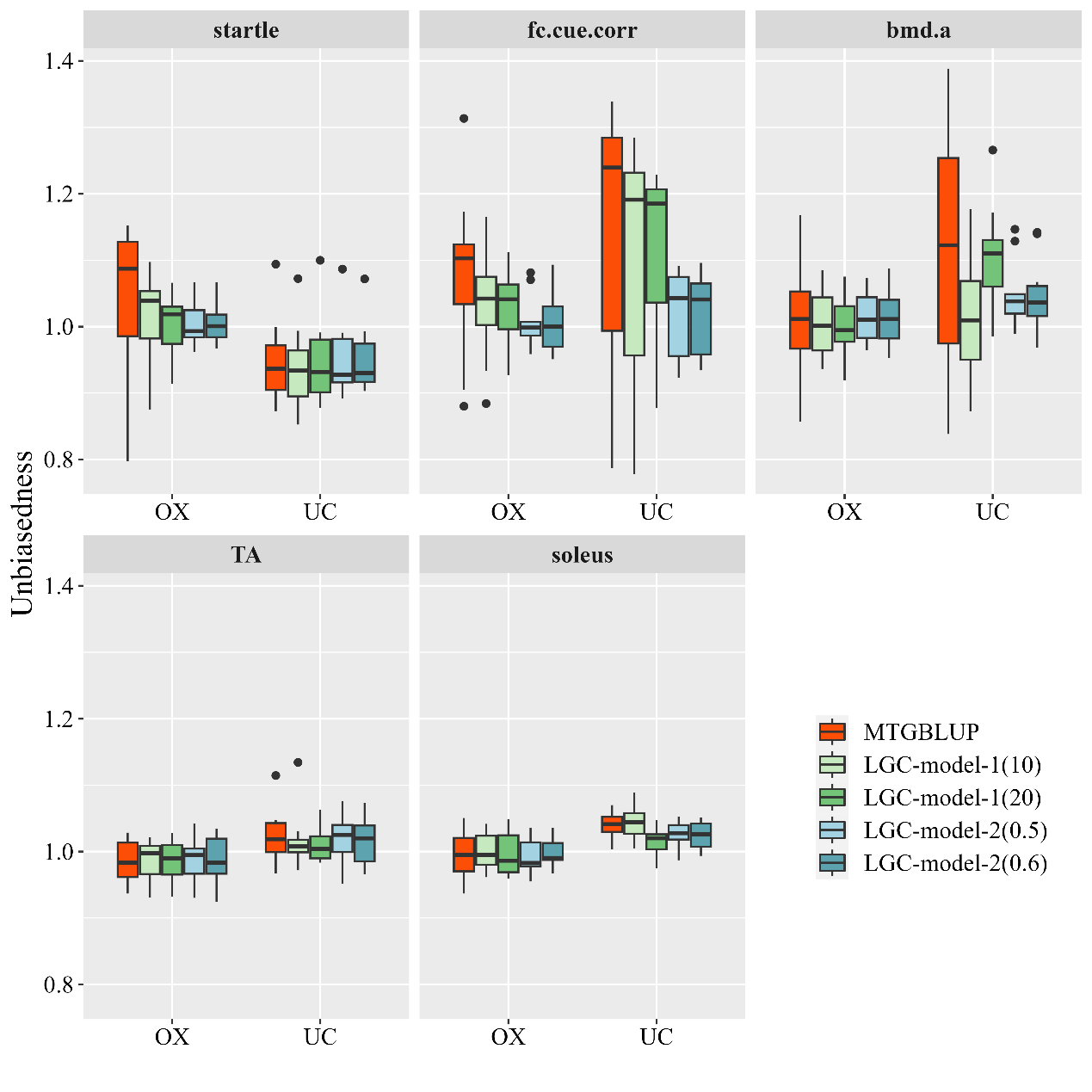


**Figure S5.** Unbiasedness of genomic prediction of the conventional multi-trait model (MTGBLUP) and the two local genetic correlation models (LGC-model-1 and LGC-model-2) in the OX and UC populations of the mice dataset. For LGC-model-1, two thresholds for significance were applied: top 10 regions (i.e., the 10 most significant regions) and top 20 regions. For LGC-model-2, two thresholds for distinguishing strong correlations were applied: $\left| \hat{r}_{lgc} \right|$ ≥ 0.5 and $\left| \hat{r}_{lgc} \right|$ ≥ 0.6. Fc.cue.corr: corrected freezing to cue. Bmd.a: abnormal bone mineral density. TA: weight of tibialis anterior. Soleus: weight of soleus.


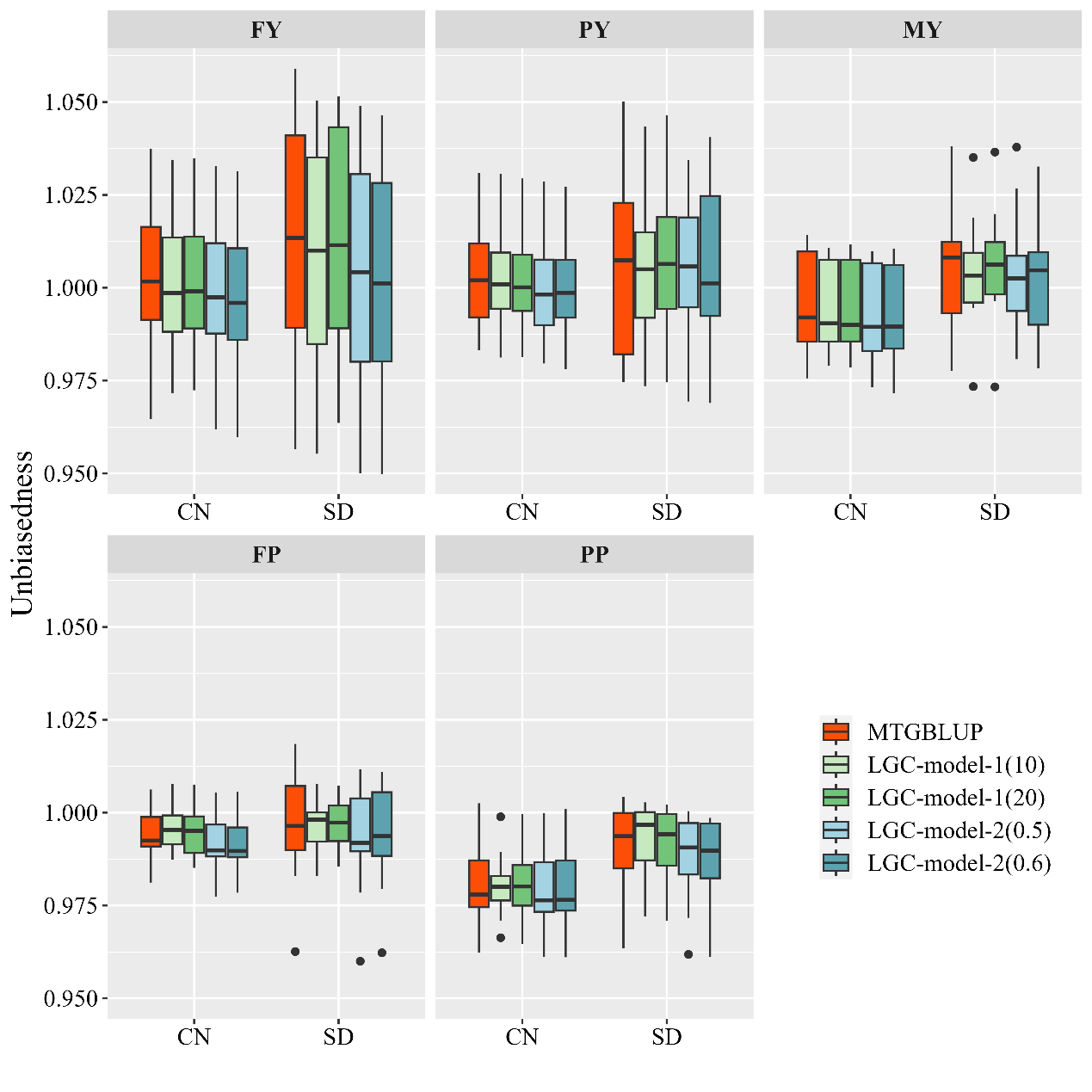


**Figure S6.** Unbiasedness of genomic prediction of the conventional multi-trait model (MTGBLUP) and two local genetic correlation models (LGC-model-1 and LGC-model-2) in the CN and SD populations of the cow dataset. For LGC-model-1, two thresholds for significance were applied: top 10 regions (i.e., the 10 most significant regions) and top 20 regions. For LGC-model-2, two thresholds for distinguishing strong correlations were applied: $\left| \hat{r}_{lgc} \right|$ ≥ 0.5 and $\left| \hat{r}_{lgc} \right|$ ≥ 0.6. FY: milk fat yield. PY: milk protein yield. MY: milk yield. FP: milk fat percentage. PP: milk protein percentage.
